# Supplementary material for: Function of Graphene Oxide as the “Nanoquencher” for Hg2+ Detection Using an Exonuclease I-Assisted Biosensor
Source: Int J Mol Sci. 2022 Jun 5;23(11):6326. doi: 10.3390/ijms23116326 (PMC9180964; doi:10.3390/ijms23116326)
Supplement: Supplementary file 1 [file ijms-23-06326-s001.zip › ijms-1744365-supplementary.pdf]

Function of graphene oxide (GO) as the "nanoquencher" for Hg^{2+} detection using an exonuclease I-assisted biosensor

Ting Sun[1], Xian Li[1], Xiaochuan Jin[1], Ziyi Wu[1], Xiachao Chen[2], Jieqiong Qiu[1,*]

[1] College of Life Sciences and Medicine, Zhejiang Sci-Tech University, Hangzhou 310018, People's Republic of China

[2] School of Material Sciences & Engineering, Zhejiang Sci-Tech University, Hangzhou 310018, People's Republic of China

*Corresponding author:

Jieqiong Qiu

College of Life Sciences and Medicine, Zhejiang Sci-Tech University, Hangzhou 310018, People's Republic of China

Email: qiujieqiongqjq@163.com; qiujieqiong@zstu.edu.cn

Tel: 0571-86843192

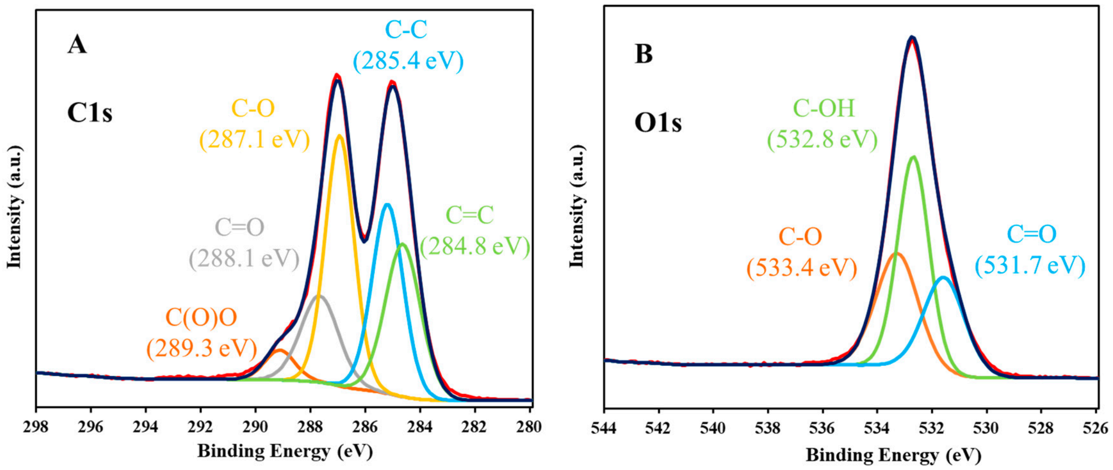

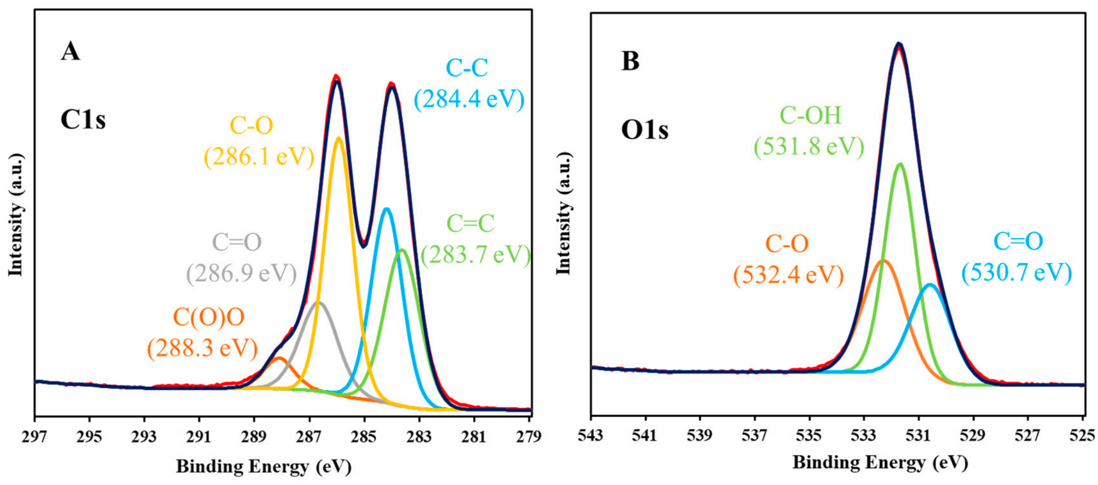

Figure S1 High-resolution XPS spectra of GO: (A) C1s spectrum, (B) O1s spectrum

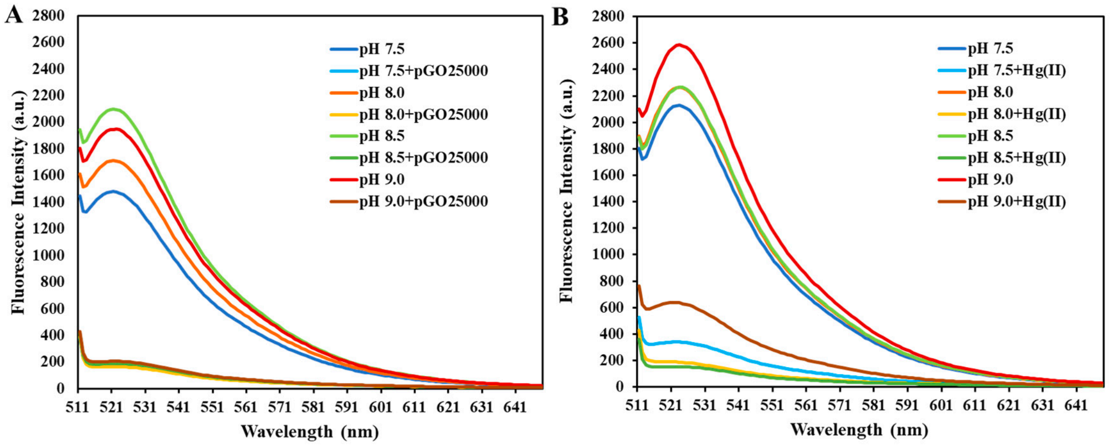

Figure S2 (A) Effect of pH values on the fluorescence intensity of FAM-ssDNA with 600 nM Hg(II) solution in the absence and presence of 0.1 μg/mL pGO25000. (B) The effect of pH values on the fluorescence intensity of FAM-ssDNA with or without 600 nM Hg(II) solution in the presence of 0.1 μg/mL pGO25000 and 2 U Exonuclease I

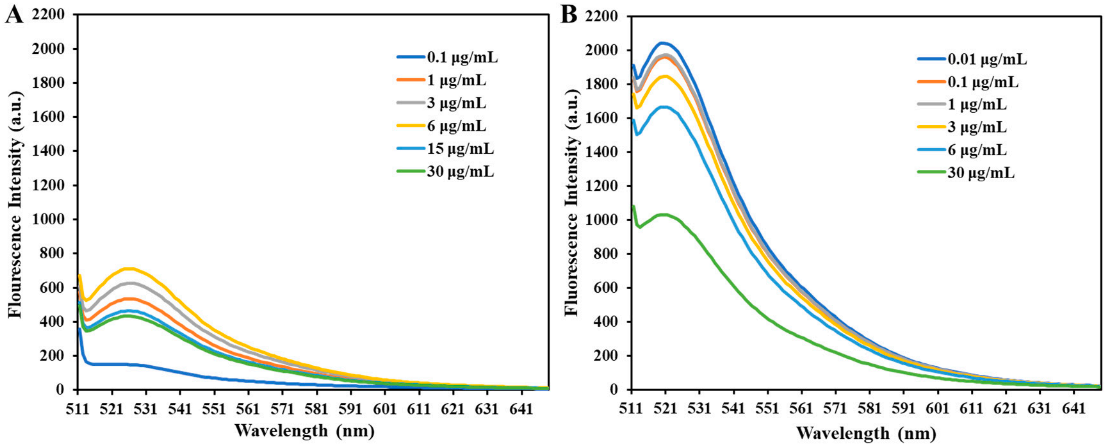

Figure S3 (A) Effects of pGO25000 concentrations from 0.1 to 30 μg/mL on fluorescence quenching of FAM-ssDNA. (B) Effects of GO concentrations from 0.01 to 30 μg/mL on fluorescence quenching of FAM-ssDNA

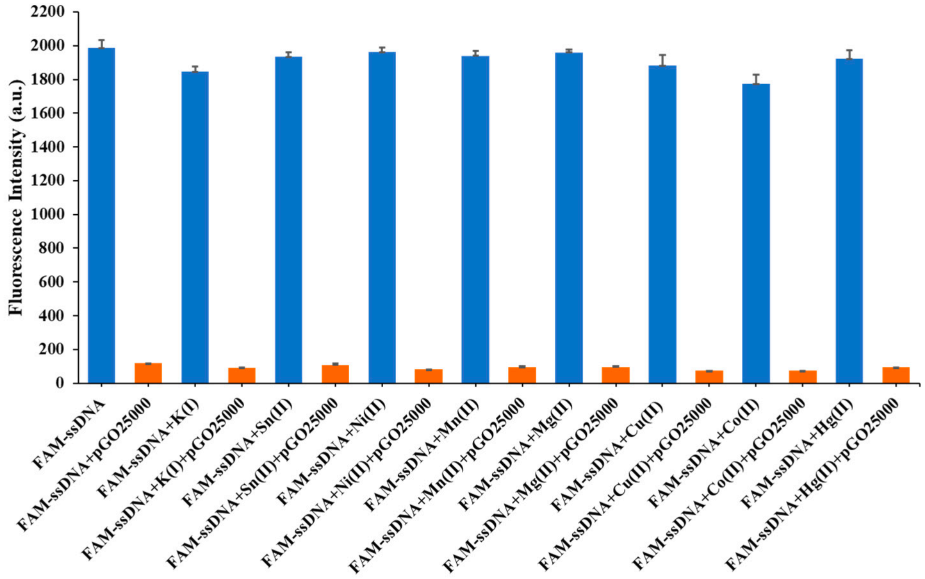

Figure S4 Selectivity of the FAM-ssDNA probes quenched by the pGO25000. The fluorescence intensities of the FAM-ssDNA probes were recorded in the absence and presence of pGO25000 (0.1 μg/mL) with the possible interference metal ions (K^{+}, Sn^{2+}, Ni^{2+}, Mn^{2+}, Mg^{2+}, Cu^{2+}, Co^{2+}, Hg^{2+}) respectively. *E*x=495 nm, *E*m=520 nm. Each sample solution was measured three times
